# Increased risk of type 3c diabetes mellitus after acute pancreatitis warrants a personalized approach including diabetes screening

**DOI:** 10.1093/bjsopen/zrac148

**Published:** 2022-12-14

**Authors:** Alexander Walker, James O’Kelly, Catriona Graham, Sian Nowell, Doug Kidd, Damian J Mole

**Affiliations:** MRC Centre for Inflammation Research, University of Edinburgh, Edinburgh, UK; MRC Centre for Inflammation Research, University of Edinburgh, Edinburgh, UK; Edinburgh Clinical Research Facility, University of Edinburgh, Edinburgh, UK; eData Research & Innovation Service (eDRIS), formerly Information Services Division, NHS National Services Scotland now part of Public Health Scotland, Edinburgh, Scotland, UK; eData Research & Innovation Service (eDRIS), formerly Information Services Division, NHS National Services Scotland now part of Public Health Scotland, Edinburgh, Scotland, UK; MRC Centre for Inflammation Research, University of Edinburgh, Edinburgh, UK; Clinical Surgery, University of Edinburgh, Royal Infirmary of Edinburgh, Edinburgh, Scotland, UK

## Abstract

**Background:**

Acute pancreatitis (AP) is a frequent cause of hospitalization with long-term health consequences, including type 3c diabetes mellitus (DM). The incidence and risk factors for new-onset morbidities after AP need to be clarified to inform a personalized medicine approach.

**Methods:**

Using a longitudinal electronic healthcare record-linkage analysis, all patients admitted to hospital in Scotland with a first episode of AP between 1 April 2009 and 31 March 2012 and followed for a minimum of 5 years after their index AP admission were identified. All new-onset morbidity with specific focus on type 3c DM were analysed and, using time-split multiple regression.

**Results:**

A total of 2047 patients were included. AP requiring critical care was followed by 2 years of heightened risk (HR 5.24) of developing type 3c DM, increased risk of new-onset cardiac disease (HR 1.61), and renal disease (HR 2.96). The additional risk conferred by critical care AP had a negative interaction with time, whereas additional risk associated with male sex and a non-gallstone aetiology was long lasting.

**Conclusion:**

Based on these findings, a personalized approach to include type 3c DM screening for a minimum of 2 years for individuals who required critical care when hospitalized with AP is recommended.

## Teaser

Increased risk of type 3c diabetes mellitus after acute pancreatitis warrants a personalized approach to follow-up that includes diabetes screening for 2 years.

## Introduction

Acute pancreatitis (AP) remains one of the most common gastroenterological causes for hospital admission in the UK^1^. The reported incidence of AP ranges between 150 and 420 cases per million per year^1^, and the incidence is increasing. AP is acute inflammation of the pancreas gland, usually triggered by gallstones or alcohol, but other aetiologies, including dyslipidaemia, trauma, endoscopic interventions, viral infections, and certain medicines are known^2–5^. AP causes acute tissue injury in the pancreas that triggers a systemic inflammatory response, which is severe enough in one in four patients to necessitate treatment in a critical care setting (high-dependency or intensive care). The overall in-hospital fatality rate is 5 per cent, which increases to 21 per cent in those needing critical care^6^.

In those individuals who survive the index episode, there are long-term consequences. Loss of function and/or cell mass of insulin-producing β-cells of the pancreatic islets leads to pancreatogenic diabetes mellitus (DM), also termed type 3c DM^7,8^. Loss of acinar cell mass, fibrosis, structural damage, including duct disruption, together lead to exocrine pancreatic insufficiency. The legacy of the systemic inflammatory response and multiple organ failure may lead to cardiovascular, respiratory, and generalized organ hypofunction. The true incidence of type 3c DM after pancreatic injury, including AP, remains unclear due to lack of standardized follow-up, mislabelling as type 2 DM, and heterogeneity in pancreatic injury^9–11^; however ,it is estimated to affect between 5–10 per cent of the diabetic population^12,13^.

In addition to intrinsic pancreatic sequelae, the concept of survivorship has been put forward, particularly in the critical care setting. Patients admitted to critical care show an increased burden of pulmonary complications, often in the context of acute respiratory distress syndrome, and survivors of severe sepsis suffer increased levels of cognitive impairment, sarcopenia, and functional disability with associated impaired quality of life^14–16^. Patients who survive critical care admission also have a higher risk of mortality that persists after discharge and apparent recovery from the acute insult^17–19^. Recently, this concept has been brought into stark focus in individuals who fail to completely recover after severe COVID-19 infection, for which the nomenclature ‘long COVID’ has been applied^20^.

It has been previously reported that early organ dysfunction affects long-term survival in patients with AP^21^, and patients who develop severe AP are at higher risk of developing other co-morbidities, including cardiovascular, metabolic, and neoplastic disease than a matched Scottish population^22^. The aim of this study was to assess the risk factors for long-term mortality and new-onset pancreatic and extra-pancreatic co-morbidities after a first episode of AP with a special focus on new-onset DM.

## Methods

### Study design

This study followed the STROBE guidelines^23^. All patients admitted to hospital in Scotland with a first episode of AP between 1 April 2009 and 31 March 2012 were identified. Long-term follow-up was undertaken of a cohort with a minimum of 5 years actual follow-up. The record linkage included the following national datasets: Scottish Morbidity Record data sets: SMR00 (Outpatient Attendance); SMR01 (General/Acute Inpatient and Day Case); SMR04 (Mental Health Inpatient and Day Case); and SMR06 (Scottish Cancer Registry). Also included were Prescribing Information System datasets, mortality data from the National Records of Scotland, and datasets from the Scottish Intensive Care Society Audit Group (SICSAG). All data points were retrieved up to 31 March 2017. eHealth data were retrieved by the electronic Data Research and Innovation Service team, formerly part of the Information Services Division of NHS Scotland and now Public Health Scotland. AP was identified from the ICD 10th Revision (ICD-10) using classification code K85. Where a patient was admitted several times during the study interval, the first episode was included as the index event. If a patient was transferred between hospitals as part of the treatment for their index admission, the data from these hospital admissions were collated as a single index admission.

All data were handled according to the Charter for Safe Havens in Scotland.

### Variables of interest

Mortality was recorded from the National Registers of Scotland Deaths as described above.

The Charlson co-morbidity index (CCI)^24^ score was calculated for each patient before and including their index admission by adding relevant major diagnostic fields for every hospital attendance in a 5-year interval before their index admission. Previously validated ICD-10 codes were used to identify relevant diagnoses^25^. Where no hospital attendances were recorded in a 5-year interval before index admission, the CCI score was recorded as 0. In a sensitivity analysis, Cox regression analyses were performed with whole cases only, and did not influence results.

Patients were deemed to have gallstone-induced AP if a diagnosis of gallstones was recorded during the index admission, or a cholecystectomy was performed at any point during the index admission or follow-up interval. The level of care and specific organ support during the index admission were derived from SICSAG datasets. Level of care was categorized as ward-level or critical care, with the latter being a composite of level 2 (high-dependency unit; HDU) and level 3 (intensive care unit; ICU) care.

A diagnosis of obesity, hyperlipidaemia, or hypertension was recorded if before and including the index admission episode, patients were assigned a relevant ICD-10 classification code. A patient was considered to have DM if any data set included an ICD-10 classification code for DM^25^, or if they were issued with a prescription for a drug used in DM as displayed in the British National Formulary (BNF).

Subgroups of medication-controlled and insulin-controlled diabetes were obtained based on prescription data. A diagnosis of metabolic syndrome was assigned to patients with any of obesity, hyperlipidaemia, hypertension, or diabetes.

Patients were considered to suffer from chronic respiratory disease if they received a relevant ICD-10 classification code or if they were prescribed bronchodilators or respiratory steroids as listed in the BNF. Patients were defined as suffering from cardiac co-morbidities if they received a relevant ICD-10 classification code for ‘Acute Myocardial Infarction’ or ‘Heart Failure’, or if they received a prescription for anti-anginal or congestive heart failure drugs as listed in the BNF.

### Statistical methods

Categorical variables were reported as counts and percentages. Continuous variables were described by either mean and standard deviation (s.d.) for normally distributed data, or median and interquartile range (i.q.r.) for non-Gaussian data. Differences in proportions were tested by the chi-squared test. Between-group comparisons were performed using analysis of variance for three or more groups with normally distributed data.

For all-cause mortality survival analysis, individuals not recorded as dead were censored at the point of record linkage on 31 March 2017. For each new-onset co-morbidity, only those individuals who did not have that co-morbidity recorded before or during their index admission were included in the denominator. Patients were considered to have a new-onset co-morbidity if a relevant code for the co-morbidity appeared during their follow-up in either hospital or prescription data, or was listed on their death certificate. A patient who died without a new-onset co-morbidity during the follow-up interval was censored at the point of death, and all other patients were censored at the point of record linkage (31 March 2017).

For survival analyses, *P* values were initially obtained for each variable using the univariate likelihood ratio test. A specific set of variables with plausible biological significance were considered for analysis: age (years), sex (male or female), AP aetiology (gallstone or non-gallstone), level of care (ward or critical care), organ support requirement (non-invasive ventilation, intubation, haemofiltration, inotropic support, or parenteral nutrition), CCI (0, 1, or 2+), presence of preadmission diagnosis of chronic pancreatitis, DM, obesity, dyslipidaemia, and hypertension. Variables with a statistically significant relationship in the univariate analysis were included in multivariate regression and used to fit a multivariate Cox proportional hazards model using backward stepwise selection with a fixed threshold value of 0.05. Where non-proportional hazards were observed, time-by-covariate interaction terms were used to fit the Cox model, using 0.5-year serial intervals. *P* < 0.050 was taken to indicate statistical significance. The Kaplan–Meier method was used to estimate survival probabilities.

All statistics were performed in R version 3.6.1 or later.

## Results

### Demographics and index admission

Between 1 April 2009 and 31 March 2012, a total of 2047 patients presented to hospital with AP. Of these patients, 382 (18.7 per cent) were treated in the critical care setting with the rest receiving ward-level care. The overall mortality rate during the index admission for AP was 4.9 per cent (*n* = 101) with a rate of 21.7 per cent (*n* = 83) in critical care admissions compared with 1.1 per cent (*n* = 18) in ward admissions. Demographic data for the cohort are presented in *Table 1*.

**Table 1:** Baseline characteristics for study population

Characteristics	Total	Index admission deaths	Follow-up deaths	Survivors	*P*
	(*n* = 2047)	(*n* = 101)	(*n* = 451)	(*n* = 1495)	
**Age, years, mean(s.d.)**	56 (18.5)	68 (14.9)	67 (16.5)	52 (17.6)	<0.001*
Sex (Male)	1020 (49.8)	50 (49.5)	252 (55.9)	718 (48.0)	0.014†
**Aetiology, presence of gallstones**	958 (46.8)	33 (32.7)	174 (38.6)	751 (50.2)	<0.001†
**Charlson co-morbidity score before admission**					
0	1745 (85.2)	73 (72.3)	312 (69.2)	1360 (91.0)	<0.001†
1	177 (12.9)	12 (11.9)	76 (16.9)	89 (6.0)	
2+	125 (6.1)	16 (15.8)	63 (14.0)	46 (3.1)	
**Preadmission co-morbidities**					
Metabolic syndrome	311 (15.2)	28 (27.7)	99 (22.0)	184 (12.3)	<0.001†
Obesity	38 (1.9)	6 (5.9)	9 (2.0)	23 (1.5)	0.006†
Dyslipidaemia	30 (1.5)	2 (2.0)	4 (0.9)	24 (1.6)	0.488
Hypertension	211 (10.3)	20 (19.8)	68 (15.1)	123 (8.2)	<0.001†
Diabetes mellitus	211 (10.3)	13 (12.9)	74 (16.4)	124 (8.4)	<0.001†
Respiratory disease	403 (19.7)	31 (30.7)	121 (26.8)	251 (16.8)	0.001†
Cardiac disease	739 (35.7)	58 (57.4)	238 (52.8)	434 (29.0)	<0.001†
Renal disease	63 (3.1)	16 (15.8)	23 (5.1)	24 (1.6)	<0.001†
Chronic pancreatitis	121 (5.9)	6 (5.9)	42 (8.5)	73 (4.9)	0.002†
**Admission type, critical care**					
High-dependency	54 (2.6)	19 (18.9)	7 (1.6)	28 (1.9)	<0.001†
Intensive care	328 (16.0)	64 (63.3)	76 (16.9)	188 (12.6)	
Critical care: 0–48 h	94 (4.6)	27 (26.7)	19 (4.2)	48 (3.2)	<0.001†
Critical care: >48 h	287 (14.0)	55 (54.5)	64 (14.2)	168 (11.2)	
Filtration	68 (8.2)	37 (36.6)	9 (2.0)	22 (1.5)	<0.001†
Intubation	130 (6.4)	60 (59.4)	24 (5.3)	46 (3.1)	<0.001†
Non-invasive ventilation	52 (2.5)	22 (21.8)	13 (2.9)	17 (1.1)	<0.001†
Parenteral nutrition	65 (3.2)	28 (27.7)	10 (2.2)	27 (1.8)	<0.001†
Inotropic support	142 (6.0)	65 (64.3)	28 (6.2)	49 (2.2)	<0.001†
Total	382 (18.7)	83 (82.2)	83 (18.4)	216 (14.4)	

Values are *n* (%) unless otherwise indicated. *Analysis of variance. †Chi-squared test.

### Follow-up and long-term mortality after index admission

**Fig. 1.**
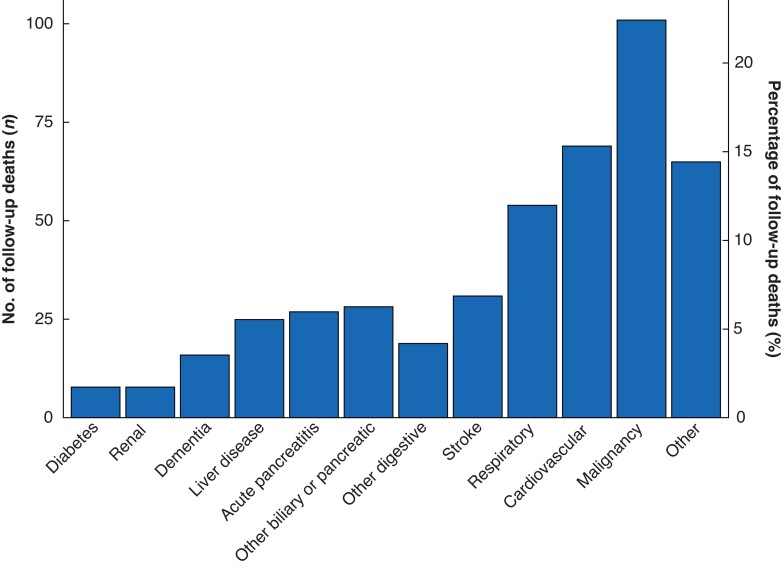
Cause of death during follow-up period. Categories derived from ICD10 diagnoses.

**Fig. 2.**
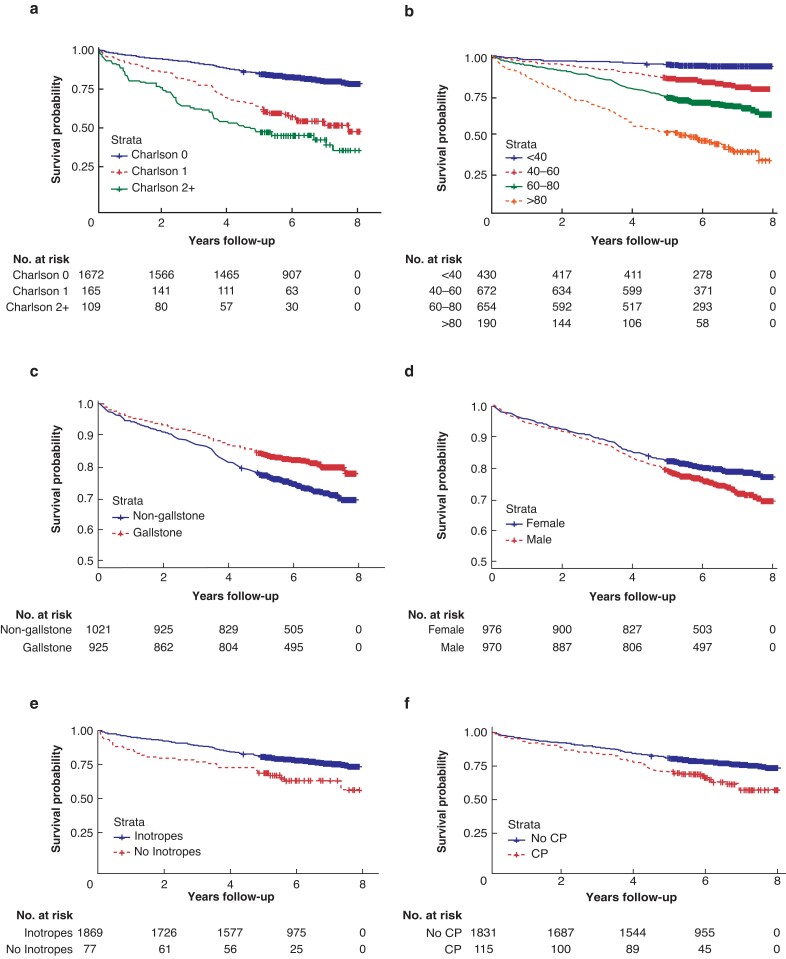
Overall survival **a** Sex. **b** Age. **c** AP aetiology. **d** Charlson co-morbidity index. **e** Inotrope requirement. **f** CP status. AP, acute pancreatitis; CP, chronic pancreatitis.

Overall, 1946 patients survived the index admission. Median follow-up after discharge was 6.1 years (i.q.r. 5.2–7.0). During this follow-up interval, 451 of 1946 patients died (23.2 per cent). *Figure 1* shows the cause of death for these patients. Multivariate regression analysis was performed to identify risk factors for all-cause mortality in those surviving the initial admission; significant risk factors included male sex, increased age, non-gallstone aetiology, and increased CCI (*Table 2*). Kaplan–Meier curves described by risk factor are presented in *Fig. 2*. Critical care admission during the index episode of AP was significantly associated with increased mortality during follow-up in univariate (HR 1.32, *P* = 0.03), but not in multivariate Cox regression.

**Table 2:** Predictors of mortality during follow-up interval after discharge for index event

Risk factor	Univariate analysis		Multivariate analysis	
	HR for death (95% c.i.)	*P*	HR for death (95% c.i.)	*P*
Age (years)*	1.05 (1.04–1.05)	<0.001	1.05 (1.04–1.06)	<0.001
Sex* (Male)	1.29 (1.08–1.56)	0.006	1.30 (1.08–1.57)	0.007
Level of care (critical care)	1.32 (1.04–1.67)	0.030	–	–
Non-invasive ventilation	2.10 (1.12–3.64)	0.009	–	–
Intubation	1.74 (1.15–2.62)	0.008	–	–
Filtration	1.44 (0.74–2.78)	0.280	–	–
Inotropic support*	1.88 (1.28–2.76)	0.001	1.63 (1.11–2.40)	0.012
Parenteral nutrition	1.27 (0.68–2.38)	0.455	–	–
Pancreatitis aetiology* (non-gallstone)	1.49 (1.23–1.82)	<0.001	1.66 (1.36–2.02)	<0.001
Chronic pancreatitis*	1.76 (1.28–2.42)	0.001	1.97 (1.51–2.51)	<0.001
Charlson co-morbidity index* (reference category = 0)	**1 **2.92 (2.27–3.75)	<0.001	1.94 (1.51–2.51)	<0.001
	**2+** 4.45 (3.39–5.84)	<0.001	3.08 (2.34–4.04)	<0.001
Metabolic syndrome	1.83 (1.47–2.29)	<0.001	–	–
Dyslipidaemia	0.61 (0.23–1.65)	0.333	–	–
Obesity	1.30 (0.67–2.52)	0.433	–	–
Hypertension	1.79 (1.38–2.32)	<0.001	–	–

Univariate and multivariate Cox analyses. *Variables with *P* < 0.05 in univariate analysis were included in the multivariate analysis and retained in the final multivariate model if they remained significant. Final model concordance 0.752 (standard error 0.011). *P* value from likelihood ratio test.

### New-onset DM following index admission

Among patients who survived the index AP admission, 198 of 1946 (10.2 per cent) had a pre-existing diagnosis of DM. New-onset DM developed in 232 of 1748 (13.3 per cent) remaining patients during the follow-up interval, 93 of which developed insulin-dependent DM (40.1 per cent of new-onset DM, 5.3 per cent of the overall cohort). In the 253 patients with AP requiring critical care, 63 developed new-onset DM (63 of 253 = 24.9 per cent), compared with new-onset DM occurring in 169 of 1495 (11.3 per cent) patients with AP not requiring critical care, HR 2.37 (95 per cent c.i. 1.73 to 3.25; *P* < 0.001) (*Tables 3*, *S1* and *S2*).

**Table 3:** Predictors of new-onset diabetes during follow-up, univariate and multivariate Cox analyses

Risk factor	Univariate analysis			Multivariate analysis		
	HR	95% c.i.	*P*	HR	95% c.i.	*P*
**DM (*n* = 1748, events = 232)**						
Sex* (male)	1.67	1.28–2.17	<0.001	1.37	1.04–1.81	0.024
Age (years)	1.01	1.00–1.01	0.153	–	–	–
Aetiology* (non-gallstone)	1.85	1.41–2.44	<0.001	1.65	1.24–2.19	<0.001
Chronic pancreatitis*	2.16	1.43–3.28	<0.001	1.63	1.07–2.50	0.024
Level of care* (critical care)	2.67	2.00–3.57	<0.001	2.37	1.73–3.25	<0.001
Non-invasive Ventilation	3.47	1.78–6.77	<0.001	–	–	–
Intubation	4.34	2.77–6.81	<0.001	–	–	–
Filtration	4.03	1.99–9.17	<0.001	–	–	–
Inotropic support	3.28	2.03–5.32	<0.001	–	–	–
Parenteral nutrition*	5.03	2.87–8.81	<0.001	2.23	1.21–4.12	0.010
Charlson co-morbidity index (reference category = 0)	**1 **1.34	0.85–2.13	0.207	–	–	–
	**2+** 0.91	0.45–1.84	0.790	–	–	–
Metabolic syndrome	1.54	1.02–2.33	0.041	–	–	–
Dyslipidaemia	1.81	0.67–4.86	0.240	–	–	–
Obesity	1.91	0.71–5.14	0.199	–	–	–
Hypertension	1.44	0.93–2.24	0.102	–	–	–
**Medication-controlled DM (*n* = 1748, events = 196)**						
Sex* (male)	1.77	1.33–2.37	<0.001	1.49	1.11–2.02	0.009
Age (years)	1.00	0.99–1.00	0.394	–	–	–
Aetiology* (non-gallstone)	1.79	1.32–2.38	<0.001	1.61	1.19–2.18	0.002
Chronic pancreatitis	1.88	1.17–3.01	0.009	–	–	–
Level of care* (critical care)	2.79	2.04–3.81	<0.001	2.05	1.40–3.00	<0.001
Non-invasive ventilation	3.67	1.81–7.45	<0.001	–	–	–
Intubation*	5.25	3.33–8.27	<0.001	2.04	1.13–3.69	0.019
Filtration	4.83	2.38–9.81	<0.001	–	–	–
Inotropic support	3.97	2.44–6.45	<0.001	–	–	–
Parenteral nutrition*	6.01	3.42–10.56	<0.001	2.01	1.02–3.97	0.043
Charlson score (reference category = 0)	**1 **1.26	0.75–2.10	0.379	–	–	–
	**2+** 0.80	0.36–1.81	0.597	–	–	–
Metabolic syndrome	1.52	0.96–2.39	0.071	–	–	–
Dyslipidaemia	2.17	0.80–5.83	0.126	–	–	–
Obesity	2.30	0.85–6.20	0.993	–	–	–
Hypertension	1.38	0.85–2.24	0.194	–	–	–
**Insulin-controlled DM (*n* = 1748, events = 93)**						
Sex* (male)	2.23	1.44–3.44	<0.001	1.75	1.11–2.76	0.017
Age* (years)	0.98	0.97–0.99	0.002	0.98	0.97–1.00	0.008
Aetiology* (non-gallstone)	2.17	1.39–2.22	<0.001	1.79	1.12–2.85	0.015
Chronic pancreatitis	2.54	1.39–4.66	0.002	–	–	–
Level of care* (critical care)	4.37	2.88–6.64	<0.001	2.68	1.56–4.62	<0.001
Non-invasive ventilation	4.80	1.95–11.83	<0.001	–	–	–
Intubation*	10.31	6.15–17.29	<0.001	2.63	1.29–5.39	0.008
Filtration	10.85	5.25–22.44	<0.001	–	–	–
Inotropic support	5.64	3.07–10.35	<0.001	–	–	–
Parenteral nutrition*	11.03	5.87–20.73	<0.001	2.50	1.15–5.43	0.020
Charlson score (reference category = 0)	**1 **1.36	0.66–2.82	0.402	–	–	–
	**2+** 1.49	0.60–3.68	0.387	–	–	–
Metabolic syndrome	0.98	0.45–2.12	0.966	–	–	–
Dyslipidaemia	2.14	0.53–8.68	0.287	–	–	–
Obesity*	3.46	1.09–10.93	0.035	3.78	1.17–12.23	0.027
Hypertension	0.75	0.30–1.84	0.522	–	–	–

* Variables with *P* < 0.05 in univariate analysis were included in the multivariate analysis and retained in the final multivariate model if they remained significant. *P* value from likelihood ratio test. DM, diabetes mellitus.

**Fig. 3.**
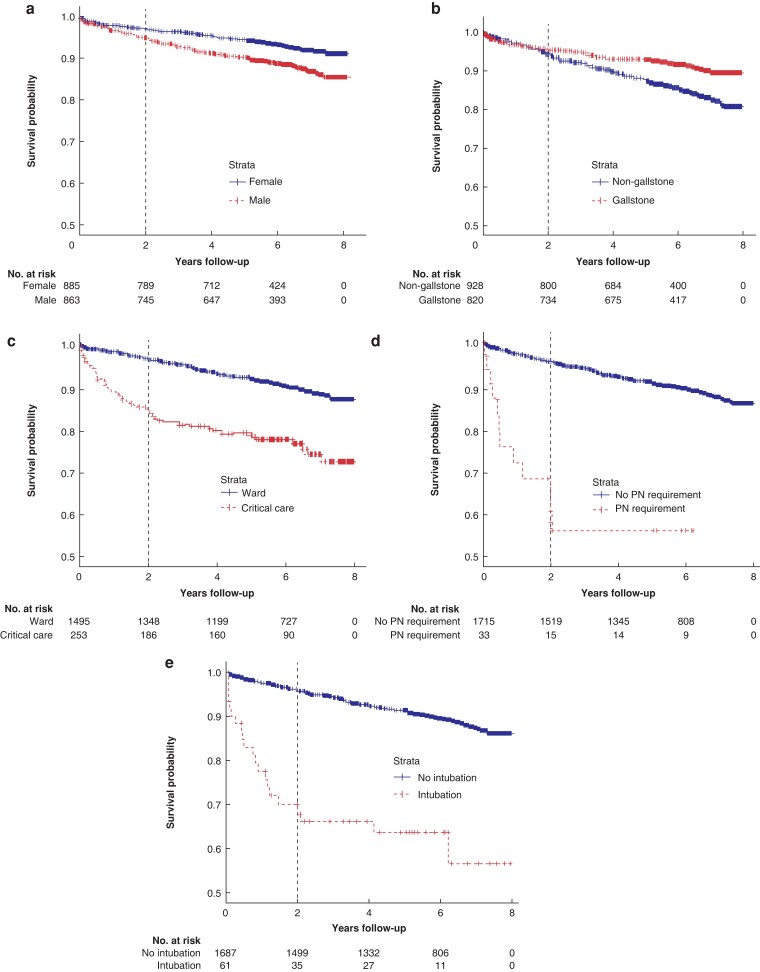
Diabetes-free survival **a** Sex. **b** Aetiology. **c** Level of care. **d** PN requirement. **e** Intubation requirement. PN, parenteral nutrition.

Male sex, chronic pancreatitis, non-gallstone aetiology of AP, critical care admission, and organ support requirement, including parenteral nutrition, were identified by univariate analysis as significant risk factors for medication- and insulin-controlled DM (*Table 3*). Initial multivariate Cox regression confirmed male sex, critical care admission, parenteral nutrition requirement ,and non-gallstone aetiology as significant risk factors for DM, with a significant change in HRs over time for both level of care and aetiology of AP. To account for these changes, time-split multiple regression was performed, using an estimated 2-year cut-off point between early and late effects based on visual inspection of the survival curves (*Fig. 3*). This confirmed a significant early effect of level of care (HR 3.19, *P* < 0.001), intubation (HR 2.65, *P* < 0.01) and parenteral nutrition (HR 2.30, *P* = 0.03), a late effect of non-gallstone aetiology (HR 2.19, *P* < 0.001), and a constant effect of male sex (HR 1.49, *P* = 0.01) on new-onset pancreatitis (*Tables 4* and *S1*). Median time from discharge to diabetes onset was 440 (i.q.r. 160–1185) days for patients with AP needing critical care, and 1115 (i.q.r. 493–1738) days for those with AP treated in a general ward setting; 627 (i.q.r. 159–1295) days for patients with gallstone AP, against 891 (i.q.r. 483–1738) days for patients with a non-gallstone aetiology of AP. Male sex, non-gallstone aetiology, and higher level of care were also significant risk factors for medication-treated diabetes and insulin-treated diabetes, with increasing age negatively associated with the development of insulin-dependent diabetes during follow-up (*Tables 4* and *S1*).

**Table 4:** Predictors of new-onset diabetes: multivariate Cox analysis

Risk factor		HR	95% c.i.	*P*
**New-onset diabetes mellitus (*n* at risk = 1748, events = 232)**				
Sex (male)	0–2 years	1.54	1.00–2.37	0.051
	>2 years	1.27	0.89–1.81	0.192
Aetiology (non-gallstone)	0–2 years	1.04	0.68–1.60	0.842
	>2 years	2.31	1.56–3.41	<0.001
Chronic pancreatitis	0–2 years	1.71	0.90–3.27	0.103
	>2 years	1.60	0.91–2.82	0.100
Level of care (critical care)	0–2 years	3.49	2.22–5.49	<0.001
	>2 years	1.70	1.07–2.68	0.026
Parenteral nutrition	0–2 years	3.16	1.56–6.38	0.001
	>2 years	0.86	0.20–3.69	0.844
**New-onset diabetes mellitus (medication-controlled) (*n* at risk = 1748, events = 196)**				
Sex (male)	0–2 years	1.59	0.99–2.35	0.053
	>2 years	1.43	0.97–2.10	0.073
Aetiology (non-gallstone)	0–2 years	1.13	0.72–1.78	0.710
	>2 years	2.19	1.45–3.33	<0.001
Level of care (critical care)	0–2 years	3.19	1.85–5.49	<0.001
	>2 years	1.48	0.85–2.57	0.170
Intubation	0–2 years	2.65	1.32–5.35	0.006
	>2 years	0.82	0.22–3.06	0.772
Parenteral nutrition	0–2 years	2.30	1.07–4.95	0.033
	>2 years	1.33	0.28–6.30	0.718
**New-onset diabetes mellitus (insulin-controlled) (*n* at risk = 1748, events = 93)**				
Sex (male)	0–2 years	1.79	0.86–3.72	0.119
	>2 years	1.67	0.94–2.98	0.083
Age (years)	0–2 years	0.99	0.97–1.01	0.197
	>2 years	0.98	0.97–1.00	0.013
Aetiology (non-gallstone)	0–2 years	0.89	0.56–2.24	0.750
	>2 years	3.16	1.59–6.26	0.001
Level of care (critical care)	0–2 years	5.52	2.29–13.32	<0.001
	>2 years	1.97	0.95–4.09	0.069
Intubation	0–2 years	3.62	1.47–8.93	0.005
	>2 years	0.84	0.17–4.13	0.828
Parenteral nutrition	0–2 years	3.43	1.39–8.42	0.007
	>2 years	0.95	0.11–8.04	0.965
Obesity	0–2 years	5.19	1.45–18.55	0.011
	>2 years	*	*	1.000

Aetiology and level of care are presented as time-by-covariate interactions to reflect non-proportionality of proportional hazards, with a cut-off of 2 years determined empirically. *Unable to calculate, insufficient patient data.

### New-onset extra-pancreatic co-morbidity after index admission

Other new-onset co-morbidities during follow-up were identified. Over the follow-up interval, 266 (16.9 per cent) developed new-onset pulmonary disease, 268 (21.0 per cent) developed new-onset cardiac disease, and 124 (6.5 per cent) patients developed new-onset renal disease during the follow-up interval. New-onset pulmonary disease was significantly associated with age (HR 1.01, *P* = 0.026), CCI score (HR 2.14, *P* = 0.006 for CCI 2+), non-gallstone aetiology (HR 1.35, *P* = 0.018), non-invasive ventilation (HR 2.31, *P* = 0.03), and preadmission obesity (HR 2.45, *P* = 0.02) (*Table S3* and *Fig. S1*). Significant risk factors for new-onset cardiac disease include increased patient age (HR 1.05, *P* < 0.001), increased level of care (HR 1.61, *P* < 0.01), and preadmission hypertension (HR 2.42, *P* < 0.001) (*Table S3* and *Fig. S2*); and significant risk factors for new-onset renal disease include age (HR 1.03, *P* < 0.0001), increased CCI score (HR 1.78 and 3.95 for CCI 1 and 2+ respectively, *P* < 0.05 for both), preadmission metabolic syndrome (HR 1.74, *P* < 0.01), and obesity (HR 2.48, *P* = 0.05) (*Table S3* and *Fig. S3*).

## Discussion

This analysis demonstrates clear risk factors for the development of new-onset co-morbidities after a first episode of AP, and in particular the presence of time-dependent risk factors for the development of type 3c DM after AP. These data underpin the proposal that all individuals who experience an episode of AP that requires treatment in a critical care setting should be screened regularly for DM for a minimum of 2 years after the index AP episode.

The inpatient case fatality in this cohort (4.9 per cent of all admitted patients) is comparable to other observational studies in similar populations and remains in keeping with trends in mortality from AP^1,6,26^. Median follow-up after discharge was 6.1 years and during this follow-up interval, the mortality rate was 23.2 per cent. Several studies have shown increased long-term mortality among intensive care patients^17,23,27^. In patients with AP, this same group has previously shown that multiorgan dysfunction syndrome, which is linked to, but not completely overlapping with severity of disease, is a risk factor for long-term mortality over a 10-year period^21^. In the present study, however, admission to critical care was only identified as a significant risk factor for death in univariate analysis, but not in multivariate analysis. This could be explained by the shorter actual follow-up, and that the multiple organ dysfunction score (MODS) > 2 used previously is qualitatively different from critical care admission, as a risk factor, despite there being significant overlap between the two.

A high incidence of DM following a first episode of AP is acknowledged in the literature^8^. Although the present analysis confirms a higher incidence of DM after AP than would be expected in the general population (annual incidence of 2525 in 100 000 in this population, compared with 320–400 in 100 000 recorded in the general population^28^), it remains lower than that reported by a meta-analysis by Das *et al.*^8^. This probably reflects the high proportion of patients with severe AP included in the meta-analysis, supporting the observation that severity of AP is a significant determinant of new-onset DM.

The main finding from this study is the identification of time-dependent risk factors for new-onset type 3c DM after AP. Admission to critical care was associated with an increased risk of DM within 2 years of discharge, whereas non-gallstone AP was associated with an increased risk of DM from 2 years onwards. These findings suggest actionable follow-up measures. There is currently no clear consensus regarding screening and treatment for patients with type 3c DM after AP. In the UK, guidelines for the management of pancreatitis suggest that patients with chronic pancreatitis, who account for 75–80 per cent of patients with type 3c DM, should undergo diabetic screening with glycated haemoglobin on a biannual basis^6^. Type 3c DM has important distinctions compared with type 1 and type 2 DM. Up to 80 per cent of patients with type 3c DM suffer both exocrine and endocrine insufficiency, and the loss of pancreatic tissue is linked to changes in inflammatory and counter-regulatory hormones after AP, including an altered glucagon secretion and changes in pancreatic polypeptide and hepatic glucose sensitivity^10,29–32^. These changes are suspected to contribute to a clinical pattern of poor glycaemic control and an early requirement for insulin therapy in patients with type 3c DM. Therefore, the identification of new-onset DM after AP is clinically relevant. This study identifies critical care admission as a clinically important risk factor for early development of DM, suggesting regular interval screening on a personalized basis, for 2 years, targeting those admitted to critical care when hospitalized with AP and especially those patients who required organ support, to specifically identify early those patients with type 3c DM and poor glycaemic control.

Age and preadmission co-morbidities estimated by the CCI, but not sex, were independent risk factors for new-onset cardiac, pulmonary, and renal disease. The CCI is a validated predictive tool using administrative databases or medical registers and has validated use for both clinical prognosis and co-morbidity adjustment^24,25,33^. Of note, although CCI score was associated with new-onset pulmonary and renal co-morbidities, as well as long-term mortality, it was not associated with the development of new-onset DM.

This study has important limitations Potentially important factors, including change in patient BMI, smoking cessation, and alcohol abstinence during the follow-up interval were not available for this analysis. CCI score was calculated on previous hospital admission, and it was not possible to retrieve data on co-morbidities that are treated in an outpatient setting. No precise MODS score and Acute Physiology and Chronic Health Evaluation II score, nor Balthazar CT severity index was available, and instead the severity of disease was taken using the surrogate of level of care during the index admission. Despite that the extent of pancreatic necrosis and islet cell destruction is likely to be an important factor in the development of new-onset DM, the administrative coding data for necrosis drainage or necrosectomy was of insufficient quality to be included in this analysis.

This record-linkage analysis of patients with a first episode of AP demonstrates independent risk factors for new-onset morbidity and long-term mortality in a Scottish national cohort. This analysis identifies admission to critical care as a clinically significant risk factor for early onset of type 3c DM. Based on these findings, a personalized approach to follow-up to include DM screening for a minimum of 2 years for individuals who required critical care when hospitalized with AP is recommended.

## Supplementary Material

zrac148_Supplementary_Data

## Data Availability

All primary data are held within the National Safe Haven with restricted access and handled according to the Charter for Safe Havens in Scotland. Please contact the senior author (D.J.M.) in the first instance for data availability requests.
