# Supplementary material for: Increased risk of type 3c diabetes mellitus after acute pancreatitis warrants a personalized approach including diabetes screening
Source: BJS Open. 2022 Dec 14;6(6):zrac148. doi: 10.1093/bjsopen/zrac148 (PMC9749482; doi:10.1093/bjsopen/zrac148)
Supplement: zrac148_Supplementary_Data [file zrac148_supplementary_data.docx]

**Supplementary Material**

Increased risk of type 3c diabetes mellitus after acute pancreatitis warrants a personalised approach including diabetes screening

**Authors**:

Alexander Walker^1^

James OKelly^1^

Catriona Graham^2^

Sian Nowell^3^

Doug Kidd^3^

Damian Mole^1,4^

**Affiliations**:

1. MRC Centre for Inflammation Research, University of Edinburgh, Edinburgh, UK
2. Edinburgh Clinical Research Facility, University of Edinburgh, Edinburgh, UK
3. eData Research & Innovation Service (eDRIS), formly Information Services Division, NHS National Services Scotland now part of Public Health Scotland
4. Clinical Surgery, University of Edinburgh, Edinburgh, UK

**Correspondence**: [alex.walker@ed.ac.uk](mailto:alex.walker@ed.ac.uk); [damian.mole@ed.ac.uk](mailto:damian.mole@ed.ac.uk)

**Supplementary Figures and Tables**

| **Table S1**. Predictors of new-onset diabetes, final cox regression model.  **Table S2.** Predictors of new-onset diabetes during follow-up, multivariate cox analysis.  **Table S3.** Predictors of new-onset comorbidities during follow-up, multivariate cox analysis.  **Figure S1** Kaplan Meyer curves with new-onset pulmonary disease as event.  **Figure S2** Kaplan Meyer curves with new-onset cardiac disease as event.  **Figure S3** Kaplan Meyer curves with new-onset renal disease as event. | *pag. 2*  *pag. 3*  *pag. 4-5*  *pag. 6*  *pag. 7*  *pag. 8* |
| --- | --- |

**Table S1**. Predictors of new-onset diabetes, final cox regression model. Significant interaction terms between exploratory variables and time indicate time-dependent hazard ratios.

| Risk Factor | HR | 95% CI | P |
| --- | --- | --- | --- |
| **Diabetes Mellitus** | | | |
| Sex (Male) | 1.37 | 1.04 – 1.80 | 0.025 |
| Aetiology (non-gallstone) | 1.06 | 0.69 – 1.63 | 0.788 |
| Level of Care (Critical Care) | 4.01 | 2.55 – 6.31 | <0.001 |
| Chronic pancreatitis | 1.64 | 1.08 – 2.51 | 0.022 |
| Parenteral nutrition | 2.26 | 1.22 – 4.17 | 0.009 |
| Aetiology : Time | 1.19 | 1.04 – 1.37 | 0.010 |
| Level of Care : Time | 0.79 | 0.67 – 0.92 | 0.003 |
| **Diabetes Mellitus (medication-controlled)** | | | |
| Sex (Male) | 1.49 | 1.10 – 2.01 | 0.009 |
| Aetiology (non-gallstone) | 1.62 | 1.20 – 2.19 | 0.002 |
| Level of Care (Critical Care) | 4.18 | 2.47 – 7.09 | <0.001 |
| Intubation | 1.90 | 1.05 – 3.43 | 0.033 |
| Parenteral nutrition | 2.03 | 1.04 – 3.99 | 0.039 |
| Level of Care : Time | 0.62 | 0.47 – 0.81 | <0.001 |
| **Diabetes Mellitus (insulin-controlled)** | | | |
| Sex (Male) | 1.70 | 1.108– 2.68 | 0.022 |
| Age (Years) | 0.98 | 0.97 – 1.00 | 0.009 |
| Aetiology (non-gallstone) | 0.68 | 0.33 – 1.39 | 0.289 |
| Level of Care (Critical Care) | 14.11 | 6.80 – 29.30 | <0.001 |
| Intubation | 2.60 | 1.27 – 5.32 | 0.009 |
| Parenteral nutrition | 2.47 | 1.14 – 5.35 | 0.022 |
| Aetiology : Time | 1.48 | 1.16 – 1.99 | 0.002 |
| Level of Care : Time | 0.61 | 0.46 – 0.80 | <0.001 |

**Table S2.** Predictors of new-onset diabetes during follow-up, multivariate cox analysis. CI confidence interval, P-value from likelihood ratio test.

|  | | *Multivariate analysis* | | |
| --- | --- | --- | --- | --- |
| Risk Factor | Events (n) | HR | 95% CI | P |
| **DM** *(n=1748, events=232)* |  |  |  |  |
| Sex* (Male) | Male 141/863  Female 81/885 | 1.37 | 1.04 – 1.81 | 0.024 |
| Aetiology* (non-gallstone) | GS 79/820  Non-GS 153/928 | 1.65 | 1.24 – 2.19 | <0.001 |
| Chronic pancreatitis * | CP 25/103  No-CP 207/1645 | 1.63 | 1.07 – 2.50 | 0.024 |
| Level of Care* (Critical care) | CC 63/253  Ward 169/1495 | 2.37 | 1.73 – 3.25 | <0.001 |
| Parenteral nutrition* | PN 13/33  Non-PN 219/1715 | 2.23 | 1.21 – 4.12 | 0.010 |
| **Medication-controlled DM** *(n=1748, events=196)* |  |  |  |  |
| Sex* (Male) | Male 122/863  Female 74/885 | 1.49 | 1.11 – 2.02 | 0.009 |
| Aetiology* (non-gallstone) | GS 68/820  Non-GS 128/928 | 1.61 | 1.19 – 2.18 | 0.002 |
| Level of Care* (Critical care) | CC 55/253  Ward 141/1495 | 2.05 | 1.40 – 3.00 | <0.001 |
| Intubation* | Intubated 21/61  Non-intubated 175/1687 | 2.04 | 1.13 – 3.69 | 0.019 |
| Parenteral nutrition* | PN 13/33  Non-PN 183/1715 | 2.01 | 1.02 – 3.97 | 0.043 |
| **Insulin-controlled DM** *(n=1748, event=93)* |  |  |  |  |
| Sex* (Male) | Male = 63/863 Female = 30/885 | 1.75 | 1.11 – 2.76 | 0.017 |
| Age* (Years) | NA | 0.98 | 0.97 – 1.00 | 0.008 |
| Aetiology* (non-gallstone) | Non-GS 65/928 GS 28 / 820 | 1.79 | 1.12 - 2.85 | 0.015 |
| Level of Care* (Critical care) | CC 36/253  Ward 57/1495 | 2.68 | 1.56 – 4.62 | <0.001 |
| Intubation* | Intubation 18/51  Non-intubated 75/1687 | 2.63 | 1.29 – 5.39 |  |
| Parenteral nutrition* | PN 11/33  Non-PN 82/1715 | 2.50 | 1.15 – 5.43 | 0.020 |
| Obesity* | Obesity 3/20  No obesity 90/2538 | 3.78 | 1.17 – 12.23 | 0.027 |

**Table S3**. Predictors of new-onset respiratory, cardiovascular and renal disease by univariate and multivariate Cox regression. HR hazard ratio, CI confidence interval. P-value reported as likelihood ratio test. *Variables with p<0.05 in univariate analysis were included in the multivariate analysis, and retained in the final multivariate model if p<0.05

|  |  | *Univariate analysis* | | *Multivariate analysis* | |
| --- | --- | --- | --- | --- | --- |
| Risk Factor |  | HR (95% CI) | P | HR (95% CI) | P |
| **New-onset respiratory disease** *(n at risk=1574, event=266)* | | | | | |
| Age* (Years) |  | 1.01 (1.00 to 1.01) | 0.020 | 1.01 (1.00 to 1.02) | 0.026 |
| Sex (Male) |  | 1.05 (0.82 to 1.33) | 0.701 |  |  |
| Level of Care (Critical Care) |  | 1.02 (0.77 to 1.43) | 0.924 |  |  |
| Non-invasive Ventilation* |  | 2.27 (1.07 to 4.82) | 0.032 | 2.31 (1.09 to 4.92) | 0.029 |
| Intubation |  | 0.78 (0.37 to 1.65) | 0.511 |  |  |
| Filtration |  | 0.53 (0.13 to 2.14) | 0.375 |  |  |
| Inotropic support |  | 0.98 (0.51 to 1.91) | 0.962 |  |  |
| Parenteral nutrition |  | 1.91 (0.90 to 4.06) | 0.090 |  |  |
| Charlson category* (reference category = 0) | 1 | 1.62 (1.02 to 2.55) | 0.040 | 1.39 (0.87 to 2.21) | 0.169 |
|  | 2+ | 2.14 (1.32 to 3.46) | 0.002 | 1.97 (1.21 to 3.20) | 0.006 |
| Metabolic syndrome |  | 1.22 (0.87 to 1.71) | 0.247 |  |  |
| Dyslipidaemia |  | 1.90 (0.84 o 4.26) | 0.121 |  |  |
| Obesity* |  | 2.45 (1.16 to 5.19) | 0.019 | 2.52 (1.19 to 5.37) | 0.016 |
| Hypertension |  | 1.18 (0.79 to 1.76) | 0.409 |  |  |
| Pancreatitis aetiology* (non-gallstone) |  | 1.30 (1.02 to 1.67) | 0.034 | 1.35 (1.05 to 1.72) | 0.018 |
| Chronic pancreatitis |  | 0.89 (0.50 to 1.54) | 0.650 |  |  |
| **New-onset cardiac disease** *(n at risk=1274, event=268) NOTE HIGHER CONCORDANCE OVERALL FOR CCS RATHER THAN INTUBATION* | | | | | |
| Age* (Years) |  | 1.05 (1.04 to 1.05) | <0.001 | 1.04 (1.04 to 1.05) | <0.001 |
| Sex (Male) |  | 1.10 (0.86 to 1.39) | 0.458 |  |  |
| Level of Care (Critical Care) |  | 1.43 (1.03 to 2.00) | 0.034 |  |  |
| Non-invasive Ventilation |  | 1.58 (0.70 to 3.55) | 0.268 |  |  |
| Intubation* |  | 1.88 (1.10 to 3.23) | 0.021 | 2.45 (1.43 to 4.21) | 0.001 |
| Filtration |  | 1.63 (0.66 to 2.51) | 0.332 |  |  |
| Inotropic support |  | 2.41 (1.38 to 4.21) | 0.002 |  |  |
| Parenteral nutrition |  | 1.76 (0.78 to 3.96) | 0.171 |  |  |
| Charlson category (reference category = 0) | 1 | 2.16 (1.43 to 3.27) | <0.001 |  |  |
|  | 2+ | 1.29 (0.66 to 2.51) | 0.453 |  |  |
| Metabolic syndrome |  | 2.69 (1.82 to 3.98) | <0.001 |  |  |
| Dyslipidaemia |  | 1.36 (0.34 to 5.49) | 0.661 |  |  |
| Obesity |  | 1.56 (0.58 to 4.19) | 0.377 |  |  |
| Hypertension* |  | 4.30 (2.66 to 6.94) | <0.001 | 2.42 (1.49 to 3.95) | <0.001 |
| Pancreatitis aetiology |  | 0.93 (0.74 to 1.19) | 0.580 |  |  |
| Chronic pancreatitis |  | 0.47 (0.24 to 0.91) | 0.026 |  |  |
| **New-onset renal disease** *(n at risk=1899, event=124)* | | | | | |
| Age* (Years) |  | 1.07 (1.06 to 1.08) | <0.001 | 1.03 (1.01 to 1.05) | <0.001 |
| Sex (Male) |  | 0.92 (0.65 to 1.31) | 0.647 |  |  |
| Level of Care (Critical are |  | 1.87 (1.23 to 2.83) | 0.003 |  |  |
| Non-invasive Ventilation |  | 0.63 (0.09 to 4 50) | 0.644 |  |  |
| Intubation |  | 1.68 (0.74 to 3.82) | 0.216 |  |  |
| Filtration |  | 2.67 (0.99 to 7.24) | 0.053 |  |  |
| Inotropic support |  | 2.16 (1.06 to 4.43) | 0.035 |  |  |
| Parenteral nutrition |  | 0.50 (0.07 to 3.55) | 0.484 |  |  |
| Charlson category* (reference category = 0) | 1 | 2.97 (1.81 to 4.90) | <0.001 | 1.78 (1.07 to 2.96) | 0.027 |
|  | 2+ | 6.77 (4.22 to 10.85) | <0.001 | 3.95 (2.45 to 6.37) | <0.001 |
| Metabolic syndrome* |  | 3.53 (2.42 to 5.14) | <0.001 | 1.74 (1.18 to 2.58) | 0.006 |
| Dyslipidaemia |  | 2.64 (0.98 to 7.16) | 0.056 |  |  |
| Obesity* |  | 2.93 (1.20 to 7.18) | 0.019 | 2.48 (1.00 to 6.15) | 0.049 |
| Hypertension |  | 3.15 (2.05 to 4.83) | <0.001 |  |  |
| Pancreatitis aetiology (non-gallstone) |  | 1.10 (0.76 to 1.56) | 0.584 |  |  |
| Chronic pancreatitis |  | 0.87 (0.38 to 1.98) | 0.739 |  |  |

**Figure S1** Kaplan Meyer curves with new-onset pulmonary disease as event. Survival curves described by age group (A), pancreatitis aetiology (B),Charlson co-mordbidity index (C), pre-admission obesity diagnosis (D) and non-invasive ventilation (NIV) requirement

**Figure S2** Kaplan Meyer curves with new-onset cardiac disease as event. Survival curves described by age group (A), intubation requirement during admission (B), and pre-admission hypertension diagnosis (C)

**Figure S3** Kaplan Meyer curves with new-onset renal disease as event. Survival curves described by age group (A), Charlson Comorbidity Index (B), presence of metabolic syndrome (C) and hypertension prior to index admission (D)
